# Discrimination and Recognition of Phantom Finger Sensation Through Transcutaneous Electrical Nerve Stimulation

**DOI:** 10.3389/fnins.2018.00283

**Published:** 2018-04-30

**Authors:** Mengnan Li, Dingguo Zhang, Yao Chen, Xinyu Chai, Longwen He, Ying Chen, Jinyao Guo, Xiaohong Sui

**Affiliations:** ^1^School of Biomedical Engineering, Shanghai Jiao Tong University, Shanghai, China; ^2^School of Mechanical Engineering, Shanghai Jiao Tong University, Shanghai, China; ^3^Shanghai Health 51 Net Technology Co. Ltd, Shanghai, China

**Keywords:** sensory feedback, TENS, just-noticeable difference, upper-limb prosthesis, phantom finger discrimination

## Abstract

Tactile sensory feedback would make a significant contribution to the state-of-the-art prosthetic hands for achieving dexterous manipulation over objects. Phantom finger sensation, also called referred sensation of lost fingers, can be noninvasively evoked by transcutaneous electrical nerve stimulation (TENS) of the phantom finger territories (PFTs) near the stump for upper-limb amputees. As such, intuitive sensations pertaining to lost fingers could be non-invasively generated. However, the encoding of stimulation parameters into tactile sensations that can be intuitively interpreted by the users remains a significant challenge. Further, how discriminative such artificial tactile sensation with TENS of the PFTs is still unknown. In this study, we systematically characterized the tactile discrimination across different phantom fingers on the stump skin by TENS among six subjects. Charge-balanced and biphasic stimulating current pulses were adopted. The pulse amplitude (PA), the pulse frequency (PF) and the pulse width (PW) were modulated to evaluate the detection threshold, perceived touch intensity, and the just-noticeable difference (JND) of the phantom finger sensation. Particularly, the recognition of phantom fingers under simultaneous stimulation was assessed. The psychophysical experiments revealed that subjects could discern fine variations of stimuli with comfortable sensation of phantom fingers including D1 (phantom thumb), D2 (phantom index finger), D3 (Phantom middle finger), and D5 (Phantom pinky finger). With respect to PA, PF, and PW modulations, the detection thresholds across the four phantom fingers were achieved by the method of constant stimuli based on a two-alternative forced-choice (2AFC) paradigm. For each modulation, the perceived intensity, which was indexed by skin indentations on the contralateral intact finger pulp, reinforced gradually with enhancing stimuli within lower-intensity range. Particularly, the curve of the indentation depth vs. PF almost reached a plateau with PF more than 200 Hz. Moreover, the performance of phantom finger recognition deteriorated with the increasing number of phantom fingers under simultaneous TENS. For one, two and four stimulating channels, the corresponding recognition rate of an individual PFT were respective 85.83, 67.67, and 46.44%. The results of the present work would provide direct guidelines regarding the optimization of stimulating strategies to deliver artificial tactile sensation by TENS for clinical applications.

## Introduction

Amputation inevitably brings huge damage to both physical and mental health for upper-limb amputees (Kejlaa, 1992). Prosthetic hands, especially myoelectric prostheses, can help the amputees regain a significant functional improvement, which leads to more independence and higher quality of daily lives. Typically, the myoelectric signal is recorded near the residual limb to estimate the user's intention, which usually employs an open-loop control strategy without meaningful information about the manipulation situation transmitting to the users. However, a bidirectional communication bridging the amputees and prosthetic hands is necessary for the dexterous movement execution (Rothwell et al., 1982). Currently, prosthesis users mainly rely on visual feedback to gain information on the operational status of the prosthesis, which leads to a significant mental burden. Sensory feedback is critical for getting body ownership which can help an amputee feel that the prosthesis is a part of his body rather than an alien tool, and its incorporation into the prosthetic hands would be helpful for better device compliance from the user (Biddiss and Chau, 2007; Marasco et al., 2011; Saal and Bensmaia, 2015). Practically, the significance of sensory feedback has been noticed ever since the 1950s (Clippinger et al., 1974), and has attracted great interest in recent years (Jiang et al., 2012; Antfolk et al., 2013b; Delhaye et al., 2016; Svensson et al., 2017).

The sense of touch originated from normal hands carries complicated and comprehensive information like shape, temperature, size and texture of objects. Manipulation over objects by prosthetic hands can be slow, stiff and non-intuitive without tactile feedback (Delhaye et al., 2016). Besides, absence of tactile sensation from original hands also contributes to the emotional disorders involving anxiety, depression for the upper-limb amputees (Saradjian et al., 2008; Østlie et al., 2011). Consequently, tactile sensation is also the key for the maintenance of emotional balance (Hertenstein et al., 2009) and mental health (Bexton et al., 1954; Gilmartin et al., 2013) after amputation.

Tactile sensory feedback for prosthetic hands could be delivered via either invasive or noninvasive methods (Saal and Bensmaia, 2015; Delhaye et al., 2016; Svensson et al., 2017). Invasive methods included implantable devices at the central and peripheral neural pathways through cortical microstimulation (Chen et al., 2014; Flesher et al., 2016), spinal-cord stimulation (Schouenborg, 2008), peripheral nerve stimulation (Ortiz-Catalan et al., 2014; Tan et al., 2014) and target sensory reinnervation (TSR) (Kuiken et al., 2007). With these invasive methods, sensations of lost fingers or palms were partly restored for some amputees (Tan et al., 2014; Graczyk et al., 2016). However, there are still some big challenges to achieve clinical viability due to various issues such as the risk of infection in surgery, biological rejection, chronic validation or electrode replacements, etc. (Lipschutz, 2017; Svensson et al., 2017).

On the other hand, the non-invasive ways were explored by using mechanical or electrical stimulation, which resulted in the corresponding mechanotactile (Ehrsson et al., 2008), vibrotactile (Antfolk et al., 2012), electrotactile (Clemente et al., 2016; D'Anna et al., 2017) or combinational feedback schemes (Clemente and Cipriani, 2014). Previous studies showed that mechanical or electrical stimulation at the skin of the residual limb evoked the phantom illusion of the amputees (Mulvey et al., 2009; Antfolk et al., 2013a; D'Anna et al., 2017), which was stated as referred sensation of the lost hand after amputation (Ramachandran and Hirstein, 1998; Louis and York, 2006). Considering its integration and programmable characteristics, transcutaneous electrical nerve stimulation (TENS) was employed to elicit phantom finger sensations, meaning referred sensation of lost fingers (Chai et al., 2015; D'Anna et al., 2017). TENS of the median and ulnar nerves by surface electrodes were reported to produce hand sensations for normal subjects (Forst et al., 2015), and referred sensations of phantom fingers or palms for amputated subjects (D'Anna et al., 2017) for a short period. These referred sensations were most paresthesia-like (D'Anna et al., 2017), and positions of the referred sensation were influenced by the electrode location and arm positions (Forst et al., 2015). In addition, the local skin sensation under the stimulating electrode could strongly influence the recognition of phantom fingers (D'Anna et al., 2017). Chai et al. (2015) characterized the induced sensory modalities by TENS of the phantom finger maps or territories (PFTs) on the skin of the residual stump, and indicated the long-term stability of these PFTs. Chen et al. (2017) further observed the phantom finger sensation by TENS in the somatosensory cortex using magnetoencephalography (MEG) functional neuroimaging technique. Therefore, TENS of the phantom finger territories (PFTs) will be a promising approach that has the advantage of a somatotopic sensation scheme and avoids necessity of surgery. However, the critical question that how discriminative the artificial tactile sensation under TENS of the PFTs remains unanswered.

Tactile discrimination of phantom finger sensation is closely associated with stimulating parameters exerted on the PFTs. In the present study, we carried out classical psychophysical experiments to systematically characterize the perceptual properties by varying pulse amplitude (PA), pulse frequency (PF), and pulse width (PW). To determine the effective parameter range, we measured the detection thresholds and upper limits which would elicit uncomfortable sensations. And then, within available parameter ranges, we further assessed the perceived intensities indexed by the indentation depth on the contralateral intact finger pulps. The just-noticeable difference (JND), also called the difference threshold, and Weber fractions were evaluated to estimate the subjects' capability to distinguish among different stimuli. Finally, the phantom finger recognition was characterized under simultaneous stimulation.

## Materials and methods

### Subjects

Ten volunteers were randomly recruited. Prior to the psychophysical experiment, an interview was first conducted to find out each volunteer's medical history, phantom limb sensations and whether they experienced phantom limb pain now or in history. In our psychophysical experiments, the participants had a unilateral forearm amputation, and remained psychologically healthy with PFTs near the stump. And then six adult forearm amputees (subjects 1–6, three males and three females, average age ± SD: 50 ± 13, years after amputation: 16.7 ± 11.5) were recruited. The other four volunteers were excluded without phantom finger sensation. One of them was with congenital forearm deficiency (Subject 7), one as a forearm amputee (Subject 8), and the other two with shoulder-level amputation (Subjects 9 and 10). All the ten volunteers were right handed before amputation, and the general information were presented in Table 1.

**Table 1:** General information for the amputated volunteers.

**Subjects**	**Cause of amputation**	**Amputation side and years**	**Daily prosthesis usage, type**	**Forearm stump length (cm)**	**Phantom limb senation, phantom limb pain^a^**	**Phantom finger**
1	Traumatic	L, 33	All day, cosmetic	16.5	Yes, 3	1–5
2	Traumatic	L, 29	All day, cosmetic	24.5	Yes, 1	1–5
3	Traumatic	R, 13	All day, myoelectric	16.5	Yes, 1	1–5
4	Tumor	R, 10	All day, cosmetic	24.5	Yes, 2–3	1–3, 5
5	Traumatic	R, 5	All day, cosmetic	16	Yes, 3	1, 2/3, 4, 5
6	Traumatic	L, 10	All day, cosmetic	23	Yes, 4	1–5
7	Congenital	L, 40	All day, cosmetic	6	No	None
8	Traumatic	R, 36	Half day, cosmetic	37	Yes, 1	None
9	Traumatic	R, 40	NONE	0	No	None
10	Traumatic	L, 15	NONE	0	No	NONE

a *Strength of phantom limb pain was graded with a visual analog scale (VAS) between 0 and 10*.

### Identification of PFTs

For all the six subjects, phantom finger sensations were evoked when certain skin regions near the residual stump were touched by a stylus pen with 4 mm in diameter. These regions were confirmed as PFTs. Subjects 1–3 and 6 possessed five independent PFTs, which was designated in the experiments as phantom digits D1–D5. Subject 5 had four independent PFTs without phantom sensation of the ring finger (D4). Subject 4 also had five PFTs, but the territories D2 and D3 could not be clearly discriminated. In order to be consistent for comparing the tactile discrimination among the six subjects, four PFTs labeled as D1, D2, D3, and D5 were investigated under TENS to produce phantom finger sensations corresponding to lost thumb, index, middle and pinky fingers, respectively.

The detailed procedures for locating PFTs were described as follows: (1) The subject sat in a wooden chair comfortably with his/her amputated stump naturally placed on the table, and then the stump skin was cleaned with alcohol wipes. The subject's eyes were covered with an eyeshade. (2) A stylus was used to touch the volar side of the residual stump skin, and the subject was required to quickly report if specific phantom finger sensations were produced or not. Then the stylus was moved to the next point until the whole volar side was covered. In the end, the sites corresponding to the same phantom finger were connected to form a PFT outline. The most sensitive point (MSP) referred to a finger pulp in each PFT was also clearly identified and marked. This whole process was repeated twice for each subject to validate the PFTs. Each process took approximately 35 min, and a break of 1–2 min was randomly given to allow the subjects to have a relax.

**Figure 1:**
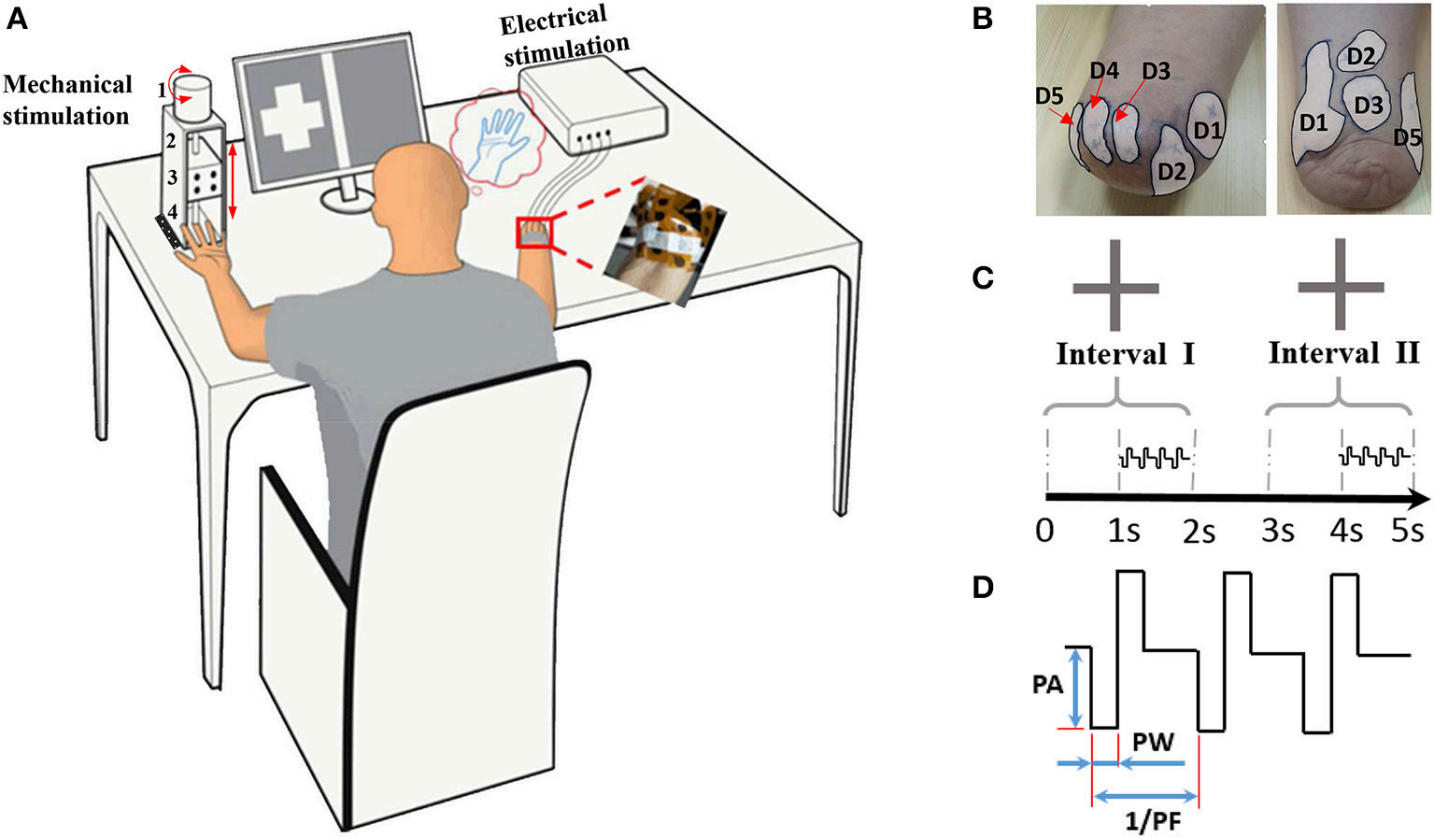
Illustration of the psychophysical experiment by TENS. **(A)** Experimental devices. 1: the step motor. 2: the ball screw 3: the moving stage. 4: the indenter; **(B)** Typical phantom finger territories near the stump for Subject 1 with D1 to D5 and Subject 4 with D1, D2, D3 and D5; **(C)** Temporal sequence of stimulating current pulses in the 2AFC paradigm for threshold determination; **(D)** Waveform schematic of stimulating current pulses. PA, Pulse Amplitude; PF, Pulse Frequency; PW, Pulse Width.

Sometimes in the procedure of identifying the PFTs, a gentle touch on the stump skin by the stylus pen only produced a local sensation of the stump skin, and the phantom finger sensations were evoked with much stronger press. The regions originated from the phantom finger pulp, back, sides, and root were all covered inside a PFT. Although skin sites referred to the phantom palm and opisthenar were also reported, these were not involved within the PFTs. Two typical PFTs were shown in Figure 1B, and the MSP was denoted as a sign “ ×,” which was considered as the TENS target location to produce the most obvious phantom finger sensation.

### Experimental devices

The current stimulator (STG 4004 stimulator, MultiChannel Systems MCS GmbH, Germany) can generate four-channel independent stimulating current pulses, which are cathodic-first, biphasic and charge-balanced (Figure 1D). The PA can be finely modulated from −16 to 16 mA with the resolution of 0.2 μA, and can hold a maximum output compliance voltage of 120 V. The PW ranges from 20 μs to infinite with a minimum interval of 20 μs. Since the pulse period can be elongated gradually from 40 μs to more than tens of hours, the corresponding PF ranges from almost zero to 25 kHz. All the stimulating parameters can be readily programmed by the control software compatible with the stimulator hardware.

To quantitatively characterize the perceived intensity of phantom finger sensation under TENS, a compact punching machine (Figure 1A) was designed to apply indentation to the contralateral intact finger pulp. The indentation depths were modulated by moving the indenter, which was a plastic rod with circular cross section mounted on a moving stage. This stage was driven by a step motor through a ball screw pair. The laptop computer was used to program the exact indentation depth, and the step precision was ±20 μm. The exact test configuration and the layout of the apparatus parts were schematically illustrated in Figure 1A.

To impose electrical stimuli on the MSP in a PFT, the flexible electrode array (Customized from Shanghai Benevolence Electronic Technology Co. Ltd., Shanghai, China) was utilized. All the electrodes were coated with a thin layer of conductive hydrogel adhesive. Two adjacent circular electrodes were defined as the stimulating and reference electrodes, respectively. Each electrode was 7 mm in diameter, and the center-to-center distance was 12 mm. The psychophysical experiments were carried out in the laboratory at 26°C.

### Experimental setup

**Figure 2:**
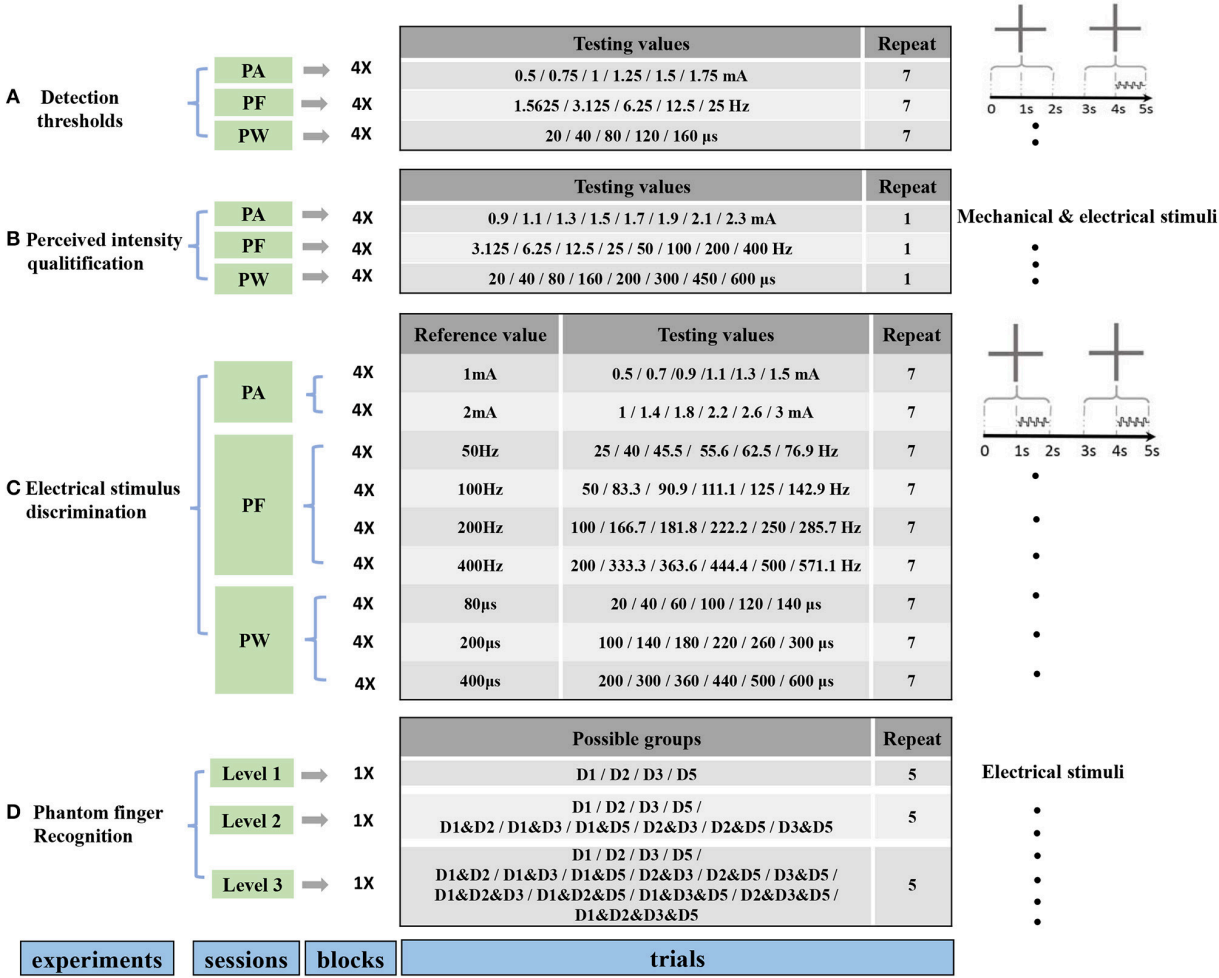
Protocols for four experiments including determination of detection thresholds **(A)**, perceived intensity quantification **(B)**, electrical stimulus discrimination **(C)** and phantom finger recognition **(D)**. The former three experiments were carried out under PA, PF, and PW modulations with four blocks corresponding to D1, D2, D3, and D5. There existed three recognition levels for phantom fingers. The stimulating trials were ordered pseudo-randomly within each block. Short breaks between trials, blocks, sessions were about 2, 30 s, 5 min, respectively.

To characterize the phantom finger sensation through TENS, a set of four experiments (Figure 2) were carried out including detection threshold determination, perceived intensity quantification, electrical stimulus discrimination and phantom finger recognition. Each experiment was divided into corresponding experimental sessions. Each session included four stimulating blocks with respect to D1, D2, D3, and D5 regions. For each block, tens to hundreds of stimulating trials were implemented. In total, there were approximately 2,200 trials for each subject. Considering the necessary breaks between trials, blocks and sessions, the whole experimental process occupied about 10 h. Thus, every subject was required to participate in these experiments twice in 2 or 3 days to maintain a relatively constant mental state.

#### Detection thresholds

The detection thresholds under TENS in each PFT were tested in terms of PA, PW and PF modulations. The procedures were double-blinded for both the experimenter and subjects. The PA, PF, and PW were set as the predetermined typical values of 1.5 mA, 50 Hz, and 200 μs, respectively. Obvious and comfortable phantom finger sensations were elicited for all the six subjects with these typical stimulating parameters. Prior to finding out the detection thresholds, the upper stimulus limits leading to an uncomfortable sensation were obtained by the method of minimal change.

Urban (1910) pointed out when determining the detection thresholds with the classical method of constant stimuli, a random stimulating order should be applied. It was also reported that the stimulus intensity must scale from the sub- and the supra- threshold values. For these reasons, it was necessary to determine the rough threshold range including both sub- and supra- thresholds in stage 1 (here by the method of limit). Based on that, the test stimuli could be further narrowed down to determine the detection thresholds with the method of constant stimuli. So the procedure was detailed into two stages including rough confirmation of threshold ranges and fine determination of detection thresholds.

In stage 1, the rough thresholds of PA, PW and PF were measured using the method of minimal change, which provided a solid basis for the selection of testing values in the fine determination of detection thresholds in stage 2. During stage 1, the stimulating pulse trains lasted 3 s. With PF at 50 Hz and PW of 200 μs, PA increased from a lower value of 0.4 mA by a step of 0.1 mA until the subject reported that the stimuli were perceived. Similarly, for rough determination of PW, PA and PF were respectively set as 1.5 mA and 50 Hz, and PW started from 20 μs with an increasing of 20 μs at each step. Also, for rough determination of PF, PF increased from 1 Hz by 1 Hz with PA and PW set as 1.5 mA and 200 μs, respectively. For four PFTs among six subjects, the rough thresholds of PA ranged from 0.6 to 1.5 mA, those of PW from 60 to 120 μs, and those of PF from 1 to 17 Hz.

On the basis of the rough threshold ranges and the output precision of the stimulator, as listed in Figure 2, the testing values in stage 2 were chosen as 0.5, 0.75, 1, 1.25, 1.5, and 1.75 mA for PA, 20, 40, 80, 120, 160 μs for PW, and 1.5625, 3.125, 6.25, 12.5, 25 Hz for PF across these six subjects, where testing values of PF decreasing from 25 Hz by 25/2^n^.

In stage 2, detection thresholds were finely determined based on the method of constant stimuli by adopting two-alternative forced-choice (2AFC) paradigm (Figure 1C), where the subject reported which of the two intervals contained the stimulus (Figure 2A). During this task, the subject was instructed to focus on two gray areas on the computer screen. Two 2-s-long stimulating intervals (Interval I first and then Interval II) were presented with 1-s break in between. Each 2-s-long interval was initiated by a centered cross in the gray area. The 1-s-long current stimuli were randomly exerted in one of the second half periods within Intervals I and II. There were no current stimuli within the first 1-s period, which helped the subject concentrate on the moment when the phantom finger sensation generated. Immediately after the disappearance of the right cross, the subject was required to report which interval contained the stimulus.

**Figure 3:**
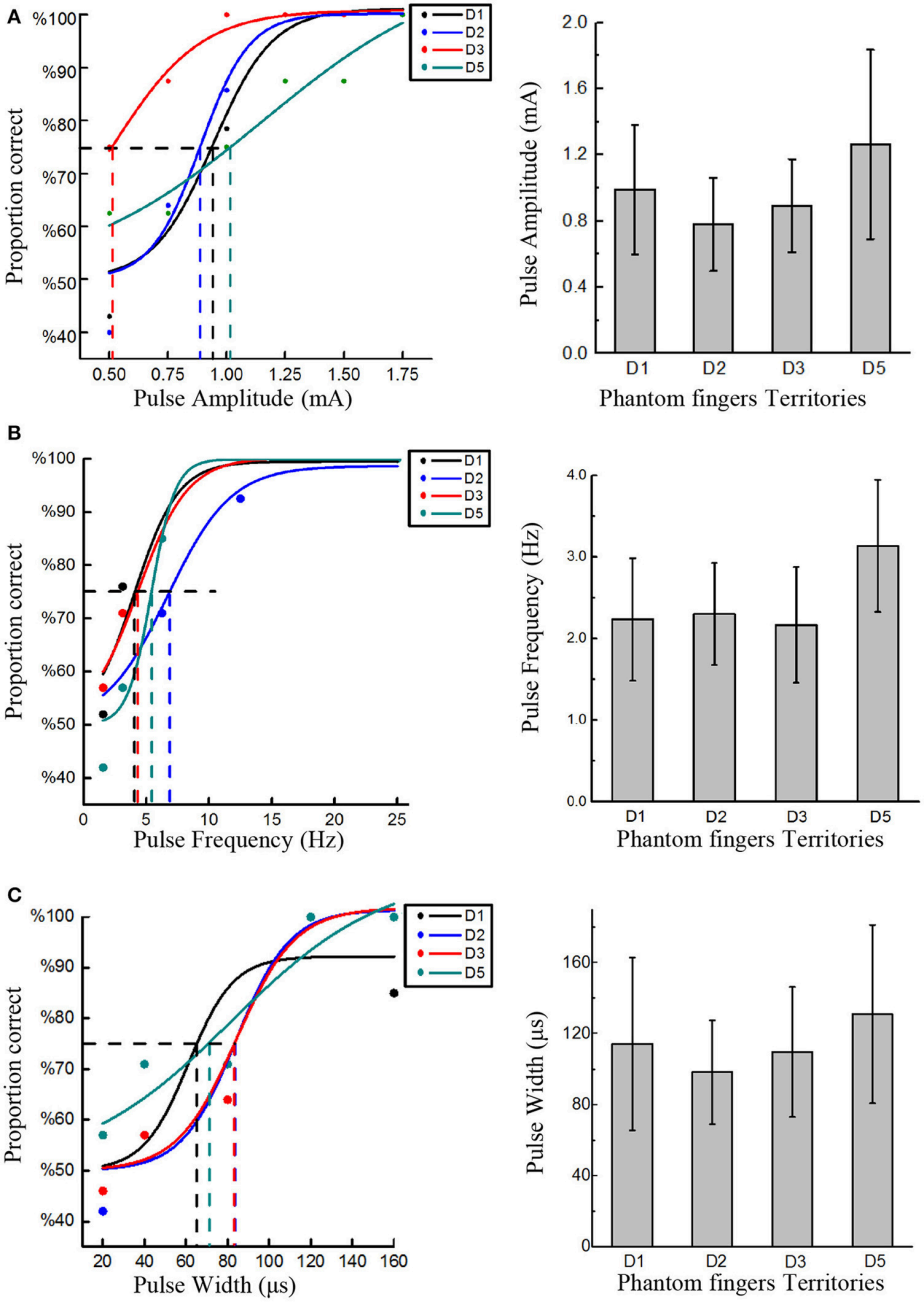
Detection thresholds under TENS of PFTs. The left three figures exemplified the method to define the detection thresholds in in PA (Subject 1), PF (Subject 2), and PW (Subject 4) modulations. The solid line was a sigmoid function of the raw data (colored dot). For 75% probability, the corresponding detection thresholds were determined. The other histogram figures in the right column showed the mean detection thresholds across four PFTs among six subjects. The detection thresholds of PA were 0.99 ± 0.39 mA, 0.78 ± 0.28 mA, 0.89 ± 0.28 mA, 1.26 ± 0.57 mA **(A)**. The mean detection thresholds in PF were 2.23 ± 0.75 Hz, 2.3 ± 0.62 Hz, 2.17 ± 0.71 Hz, 3.13 ± 0.81 Hz **(B)**. The mean detection thresholds in PW were 114.3 ± 48.75 μs, 98.3 ± 29.30 μs, 109.67 ± 36.61 μs, 131 ± 50.30 μs **(C)**.

Four stimulating blocks were presented in terms of four PFTs. Within each block, each trial was repeated 7 times for PA, PW and PF modulations, respectively, and the stimulus order within these two intervals in one trial was pseudo-randomized. Then the responses to every trial in each block were fitted by a sigmoid function. Within each trial during stage 2, the expected probability of correct judgment was 50% if the subject did not detect the stimuli at all, or otherwise the probability would rise to 100% if the subject readily detected the stimuli. Therefore, the detection thresholds were defined as the values of PA, PW, and PF that each subject could correctly identify 75% of the stimuli (Figure 3). The same criterion was also employed for intracortical sensory feedback (Flesher et al., 2016), and the probability of reaching this rate by chance was about 13.7% in our experiments.

#### Perceived intensity quantification

The PA, PW, and PF are the three common stimulus parameters which can be independently manipulated to introduce sensory feedback. In the previous work (Chai et al., 2017), multiple sensory modalities were produced by varying these three parameters. And here, we investigated the effects of these three parameters on the perceived intensities. Charge-balanced and cathodic-first stimulating current pulses were adopted in our psychophysical experiments, and variations in both PA and PW also led to changes of charge per phase. And then the indentation depth as a function of the charge per phase was further explored.

During the perceived intensity quantification, the finger being mechanically pressed on the healthy hand matched the phantom digit being tested. For example, when we applied TENS of D1, the contralateral thumb was mechanically pressed. The mechanical apparatus was kept stable on the table. The ball screw transferred the rotational displacement of the step motor into the linear displacement of the stage. The indenter protruded from the stage, and exerted the pressure on the finger pulp. There was enough space to put any of fingers between the indenter and the baseplate of the punching machine. The subject put their fingers in the baseplate axially below the indenter in a relaxed state. At point zero, there was no gap between fingers and the baseplate. The subjects could need to adjust the hand gesture to make sure that the finger pulps were in a relaxed state without introducing pre-stress in fingers. As such, the subject could readily judge the pressure intensity.

The perceived intensity of phantom finger sensation during TENS was quantitatively estimated by comparison with the indentation depth in the contralateral intact finger pulp. Every trial consisted of a 3-s-long constant current pulse train followed by a mechanical indentation. Immediately after a 3-s-long pulse train was applied into a PFT, the mechanical pressure was exerted on contralateral intact finger pulp through the indenter controlled by the punching machine shown in Figure 1A. The indentation depth was finely modulated until the perceived intensity matched to that of the electrical stimulation, and then the indentation depth was recorded. The stronger the phantom finger sensation, the deeper the indentation depth in the healthy counterpart finger. Consequently, the indentation depth was considered to be closely related to the perceived intensity of phantom finger sensation. The perceived intensities or the indentation depths were quantified in correspondence with PA, PF, and PW. Taking account of the detection thresholds, the testing values during perceived intensity quantification were listed in Figure 2B with PA, PF, and PW as typical values. The stimulating trials within each block were ordered randomly for every stimulating parameter.

Specifically, the modulation procedure of the indentation depth was further elaborated here. The position that the subject first detected the pressure was set as zero position. Then the depth increased from 0 with a step of 0.2 mm until the subject indicated that the mechanical intensity stronger than that of the electrical stimulation, and then was reduced by a step size of 0.04 mm until another reversal.

#### Electrical stimulus discrimination

The capability for a subject to discriminate the difference of stimuli is very important for artificial sensory feedback. The JNDs, also called difference thresholds, were adopted to characterize the capability to discriminate PA, PW, and PF based on the 2AFC paradigm. Similar to the determination of detection thresholds, two intervals appeared within each trial. Two 1-s-long current pulse trains, called respectively reference and test stimuli, were applied within the second half periods of these two intervals as shown in Figure 2C. The participant was requested to report the exact interval where a stronger sensation occurred. Within one trial, the two stimulating pulse trains constituted a reference/test stimuli pair and only differed in one parameter among PA, PW, and PF, with the other two fixed at the typical values.

For PA discrimination, PW and PF were held as 200 μs and 50 Hz, respectively. The reference PAs were 1 mA and 2 mA. The test PAs were set as 50, 75, 90, 110, 125, 150% of the corresponding reference values.

For PW discrimination, PA and PF were held as 1.5 mA and 50 Hz, respectively. The reference PWs were chosen as 80, 200, and 400 μs. Considering a precision step of 20 μs, the test PWs for the reference 80 μs were 20, 40, 60, 100, 120, and 140 μs. For the other two reference PWs, 50, 75, 90, 110, 125, 150% of the reference values were selected as test stimuli for PW discrimination.

For PF discrimination, PA and PW were held as 1.5 mA and 200 μs, respectively. The reference PFs were defined as 50, 100, 200, and 400 Hz, and the test PFs were approximately 50, 75, 90, 110, 125, 150% of the reference counterparts. Since the pulse frequency PF was achieved by modulating the pulse period (1/PF), so the nearest frequencies to achieve these reference percentages were used. For example, for the 50 Hz reference, the test values were 25, 40, 45.5, 55.6, 62.5, and 76.9 Hz (Figure 2C).

During the electrical stimulus discrimination, each trial was repeated 7 times within one block. Both the order of a reference/test stimulus pair and the stimulus order within the pair were pseudo-randomized and double-blinded for both the experimenter and the subjects in each block.

#### Phantom finger recognition

The experiment of phantom finger recognition was carried out in three levels with typical stimuli, i.e., PA of 1.5 mA, PW of 200 μs and PF of 50 Hz. The participant was required to point out which phantom finger or fingers were perceived. Figure 2D showed the stimulating combinations for phantom finger recognition. For Level 1, only one phantom finger was under TENS with D1, D2, D3, and D5 as the possible stimulating sites. For Levels 2 and 3, two or four PFTs at most were under simultaneous electrical stimulation to test the subjects' recognition ability of an individual PFT, and there were respectively 10 or 15 possible PFT grouping combinations. So the chance levels were 25, 10, and 6.7% for Levels 1, 2, and 3, respectively. Each trial repeated five times, and the stimuli were applied randomly in each block and double-blinded for both the experimenter and the subjects. Only a short-time stimulation less than 3 min was applied to assist the subjects' familiarization with the experiments as to Levels 2 and 3. There was no special training provided for multi-digit identification.

## Results

### Detection thresholds

**Figure 4:**
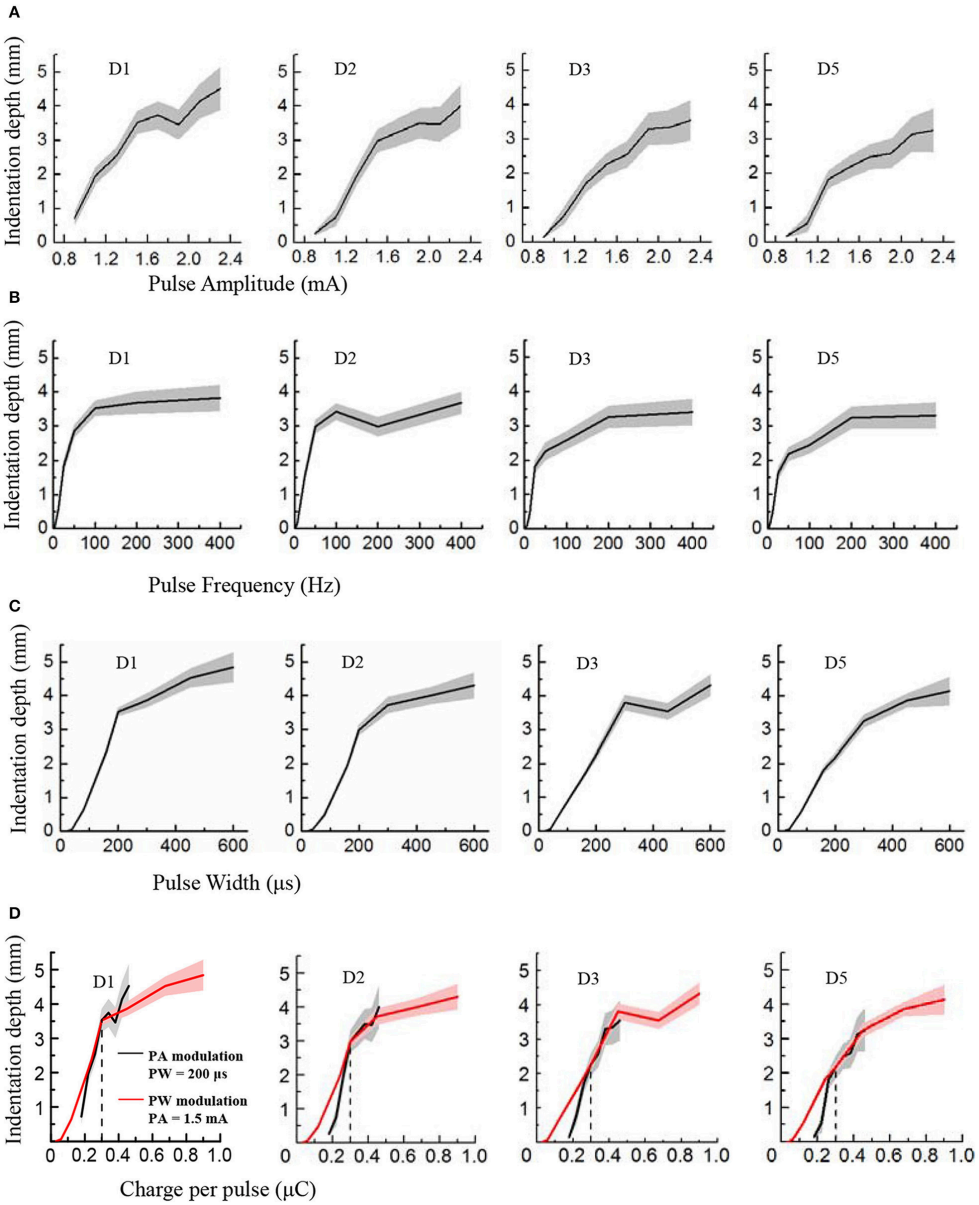
Perceived intensity quantification indexed by the indentation depth in PA, PF and PW modulations across four phantom fingers among six subjects. The solid lines and shaded regions denoted the mean and standard deviation values. **(A)** Indentation depth vs. PA; **(B)** Indentation depth vs. PF; **(C)** Indentation depth vs. PW; **(D)** Indentation depth vs. Charge per phase. Black for PA modulation with constant PW of 200 μs, and Red for PW modulation with constant PA of 1.5 mA. The vertical dotted line at 0.3 μC indicated the same indentation depth with common parameters (1.5 mA × 200 μs = 0.3 μC) under PA and PW modulations.

The rough upper limits to induce uncomfortable sensation were about 3 mA, 400 Hz, and 600 μs for PA, PW, and PF, and the detection thresholds were much lower than these upper limits. Figure 3 clearly showed the detection thresholds across six subjects. The PA detection thresholds (with PF and PW as typical values) across D1, D2, D3, and D5 were 0.99 ± 0.39 mA, 0.78 ± 0.28 mA, 0.89 ± 0.28 mA, 1.26 ± 0.57 mA, respectively. The PF detection thresholds (with PA and PW as typical values) were 2.23 ± 0.75 Hz, 2.3 ± 0.62 Hz, 2.17 ± 0.71 Hz, and 3.13 ± 0.81 Hz, respectively. The PW detection thresholds (with PA and PF as typical values) were 114.3 ± 48.75 μs, 98.3 ± 29.30 μs, 109.67 ± 36.61 μs, and 131 ± 50.30 μs, respectively. Since 200 μs and 1.5 mA were assigned to PA and PW modulations, respectively, the thresholds in terms of charge per phase were correspondingly calculated as 0.178–0.252 μC for PA and 0.147–0.195 μC for PW adapted from Figures 3A, 4C. The averaged charge threshold for PA was 0.215 μC which was moderately greater than 0.171 μC for PW. The One-way ANOVA analysis results indicated that the four PFTs had no significant difference on the detection thresholds (*P* > 0.05).

### Perceived intensity quantification

During TENS of the PFTs, the subjects experienced a wide range of perceived intensities indexed by the indentation depth in the contralateral intact finger pulps. Figure 4 illustrated that the indentation depth increased with enhancing electrical stimulus. The curves of the indentation depths vs. stimuli were basically in compliance with Steven's power function about the perceived intensity (Stevens, 1957). For lower stimuli, the slopes of curves were much steeper than those of the stronger stimuli. In the cases of PA and PW modulations, the depth boosted gradually with the advancing stimulus (Figures 4A,C). By comparison, the depth advanced much slower with PF of larger than 200 Hz (Figure 4B). What's more, the subject described the sensation in the low frequency below 10 Hz as “clearly but very slightly” corresponding to a very low indentation depth. When considering the relationships between the indentation depth and the charge per phase, Figures 4A,C were replotted in Figure 4D. The perceived intensity demonstrated a linear correlation with the enlarging charge in each phase. Especially, for charges from 0.2 to 0.5 μC in Figure 4D, the tendencies associated with PA and PW modulations matched well.

In Figure 4, the plots of the indentation depth vs. PA did not reach zero. The reason was that the lowest amplitude for PA modulation for this experiment was 0.9 mA, and an obvious perception was produced for the perceived intensity quantification experiments. So there was no zero for the indentation depth in terms of PA modulation. While for the PW modulation, the indentation depth reached zero since no perception was produced as to 20 and 40 μs, and the perception appeared under PW of 80 μs as listed in Figure 2. Moreover, at 3.125 Hz, there was still some gentle perception induced from the TENS of PFTs, and thus the indentation depth did not reach zero either.

In terms of the operational definition about detection thresholds, the subjects still had a probability of less than 75% to perceive the subthreshold stimulation. Different from this definition, the subjects definitely knew that there would be a stimulus applied to the PFTs during the experiments of perceived intensity quantification. Consequently, the subjects could perceive the electrical stimulation under small stimulus intensities. This could be the main reason why there was some difference between the lowest values in Figure 4 and the detection thresholds in Figure 3.

**Figure 5:**
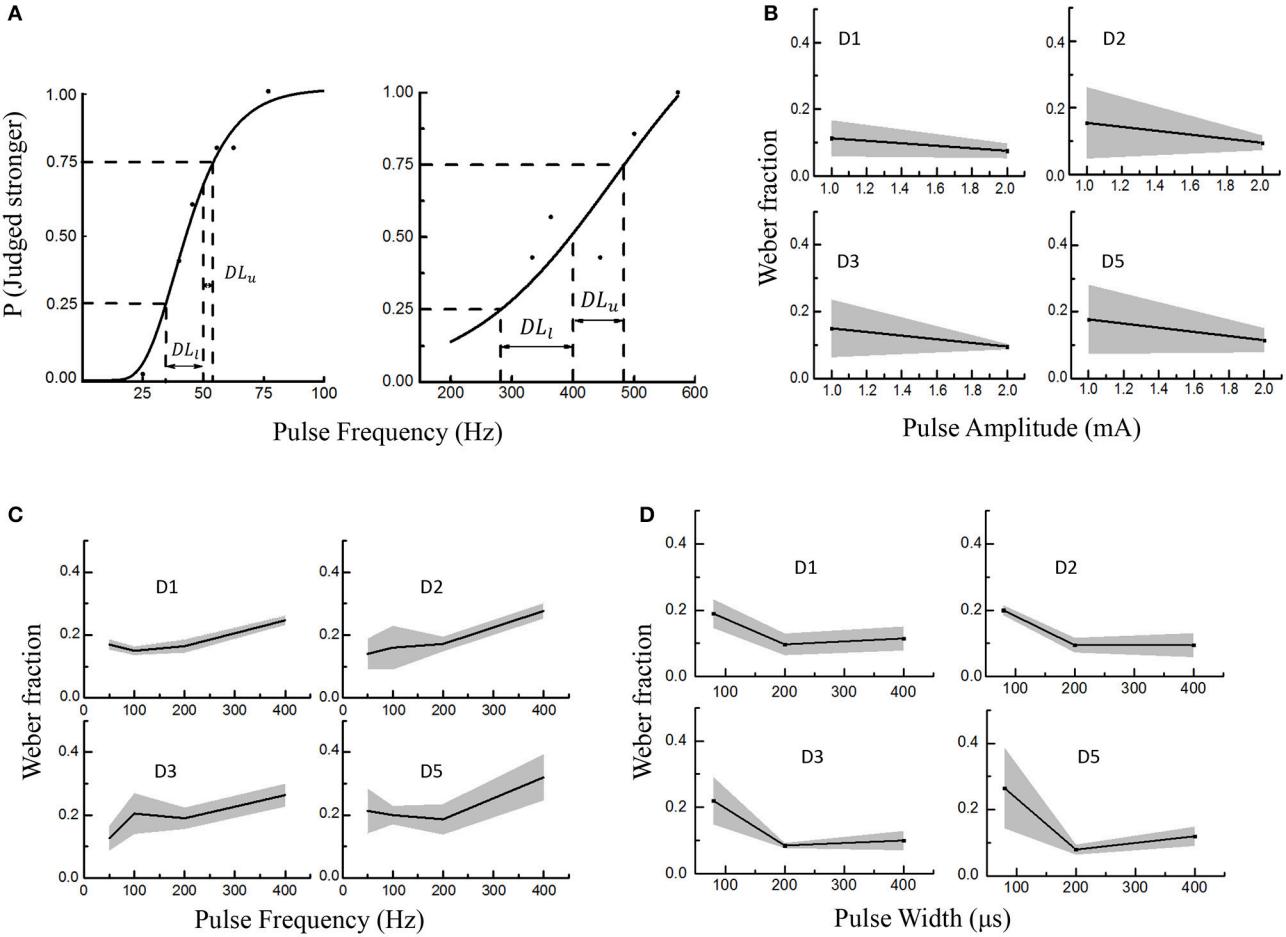
Electrical stimulus discrimination in PA, PF, and PW modulations across four PFTs involving six subjects. The solid lines and shaded regions indicated the mean and standard deviations, respectively. **(A)** Two examples as to getting the just-noticeable difference (JND) in PF (D2 in Subject 1). The reference PFs were 50 Hz (left) and 400 Hz (right). A sigmoid curve was fitted and upper and lower limits on the probability function were defined as 25% and 75% probability of correctly identifying the stronger stimulus. At last, the JND was calculated by averaging the *DL*_*l*_ and *DL*_*u*_. **(B)** The Weber fraction vs. PA; **(C)** The Weber fractions vs. PF; **(D)** The Weber fractions in PW modulation.

The plateau in the plots in Figure 4B indicated that the perceived intensity would not change much beyond a high frequency such as 100 or 200 Hz. Practically, the perceived intensity was still advanced for the high frequency. However, the discrimination deteriorated correspondingly, which was further observed from the plots in Figure 5C that the Weber fraction increased gradually beyond 200 Hz. As a result, a typical sigmoid curve appeared for 50 Hz in the JND experiment, and the discrimination data did not fit a sigmoid very well for 400 Hz as shown in Figure 5A.

### Electrical stimulus discrimination

During this experiment, the subjects were required to judge whether the test or reference stimulus was stronger within every trial. Responses by participants were converted into a probability value based on their accuracy of identifying the correct interval with the stronger stimulus. A sigmoid was fitted and upper and lower limits on this probability function were defined as 25% and 75% probability of correctly identifying the stronger stimulus. For a given reference stimulus, the JND was yielded by averaging *DL*_*u*_ and *DL*_*l*_ in Equation (1).

$$\begin{array}{r} \text{JND}=\frac{\left(DL_{u}+\;DL_{l}\right)}{2} \end{array}$$

As shown in Figure 5A, the *DL*_*u*_ and *DL*_*l*_ respectively denoted the differences between the reference stimulus with the upper limit (*L*_*u*_) and lower limit (*L*_*l*_) of the discriminated test stimuli. Figure 5A showed two curves illustrating how to define the JND for the PF modulation. To investigate the stimulus discrimination of detectable and comfortable PA, PF and PW stimuli, the Weber fraction (Ekman, 1959) was computed as shown in Equation (2).

$$\begin{array}{r} Weber\;fraction=\;\frac{JND}{reference\;stimulus} \end{array}$$

In this experiment, the average Weber fractions ranged from 0.11 to 0.18 for the PA modulation, 0.14–0.32 for PF, and 0.1–0.265 for PW. The relationships of Weber fraction with different stimuli were plotted in Figure 5 across D1, D2, D3, and D5 PFTs for six subjects. For PA and PW modulations, the Weber fractions were usually lower than 0.2, and decreased with enhancing stimulus. For PF modulation, the Weber fractions were a little larger and slightly increased within available frequency range. According to Weber's law, the Weber fraction was approximately considered as constant (Kandel et al., 2012), but this rule was not applicable for the low and high intensities with a given stimulus range (Gescheider, 1997). Here in this experiment, both 1 mA in PA and 100 μs in PW were considered as low intensities and 400 Hz in PF as the high frequency. For low intensities of PA and PW, it was sometimes very hard for some subjects to judge whether the test or reference stimulus was stronger.

By ignoring low intensities of 1 mA and 100 μs, and high intensity of 400 Hz, the proposed “optimal range” of the stimuli, which elicited a clearly discriminative sensation without uncomfortable feeling such as pain, were 1.2–2.8 mA in PA, 10–350 Hz in PF and 150–600 μs in PW. And then the corresponding Weber fractions were defined as 0.1 in PA, 0.2 in PF and 0.1 in PW.

### Phantom finger recognition

**Figure 6:**
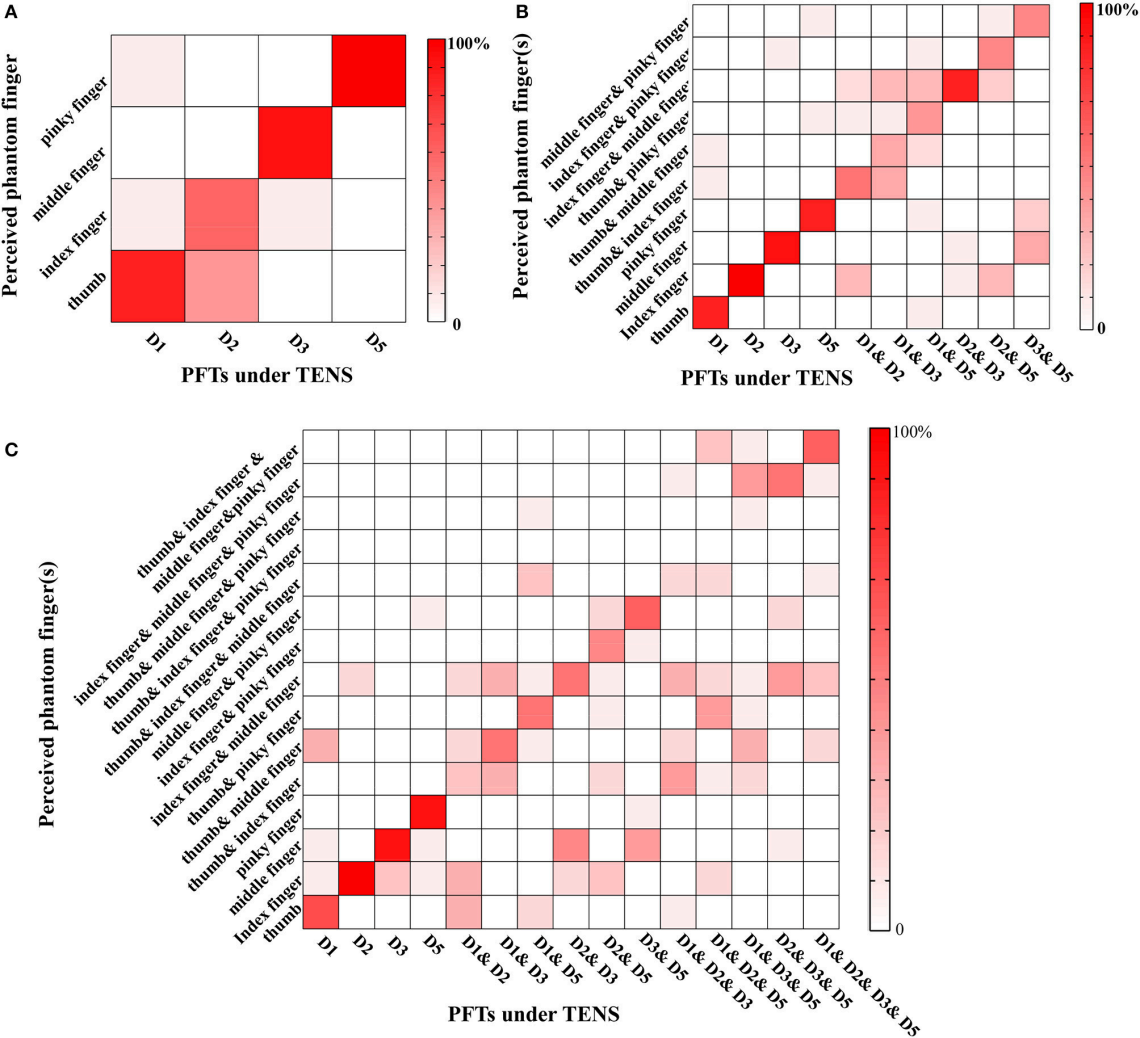
Phantom finger recognition corresponding to three levels. The horizontal and vertical axes represented the PFTs under the stimulation and the perceived phantom fingers, respectively. **(A)** Level 1 (chance level: 25%): only one PFT at most was under electrical stimulation; **(B)** Level 2 (chance level: 10%): two PFTs at most were under simultaneous TENS; **(C)** Level 3 (chance level: 6.7%): four PFTs at most were under simultaneous TENS.

The recognition performance of different PFTs was assessed in terms of three levels with typical values of PA, PF, and PW. The more the possible number of PFTs under simultaneous stimulation, the poorer the recognition performance of the individual PFT. For Levels 1–3, the correct recognition ratios about individual PFTs were 85.83% (103/120) (chance level: 25%), 67.67% (203/300) (chance level 10%), and 46.44% (209/450) (chance level 6.7%), respectively. For Level 1 (Figure 6A), the leading incorrect justice was produced due to the sensation influence from the adjacent phantom fingers (16/120). In Level 2 (Figure 6B), the misjudgments were classified into three types. The first type was the incomplete judgment (41/300). Only one phantom finger was correctly identified with two PFTs under simultaneous TENS, e.g., D1 and D2 under TENS were identified as only phantom index finger. The second was the excessive judgment (12/300). Sensation of two phantom fingers were reported with only one PFT under TENS, e.g., phantom thumb and index fingers were reported with D1 under TENS. The third was mixed with both incomplete and excessive judgments (43/300). One of two PFTs under simultaneous TENS was identified correctly but the other was misjudged as another PFT, e.g., D1 and D3 under TENS were reported as phantom thumb & index fingers. For Level 3 (Figure 6C), when TENS was applied to four PFTs at most, there were more misjudgments which were also classified as incomplete judgment (142/450, excessive judgment (33/450), and mixed misjudgment with both incomplete and excessive judgments (62/450). There were very few reports that none of the phantom fingers was identified correctly in more-than-one PFTs stimulation (5/510).

## Discussion

Our normal hand is so dexterous, with 27 degrees of freedom. Hand muscles are innervated by thousands of afferent nerve fibers which convey different (sometimes overlapping) information about objects under manipulation (Abraira and Ginty, 2013; Saal and Bensmaia, 2014). For prosthetic hands, restoring tactile feedback requires multiple stimulating channels to convey adequate information that causes appropriate tactile discrimination in association with detection and interpretation of those stimuli. Kandel et al. (2012) and Saal and Bensmaia (2015) also denoted that stimulating location and perceived intensity were critical attributes for encoding the tactile information for a specific channel and pattern coding of united activities in several channels. In addition, the existence of referred sensations near the stump about phantom limb (PL) (Hunter et al., 2008), phantom hand (PH) (Anani and Körner, 1979), and phantom finger (PF) (Björkman et al., 2016) provided a good pathway to realize artificial tactile feedback. Consequently, our present work characterized the discriminability of the perceived intensity and phantom fingers under TENS in PA, PF, and PW modulations. Four experiments were carried out including detection thresholds, perceived intensity quantification, electrical stimulus discrimination, and phantom finger recognition.

The purpose of our experiment for the detection threshold was to determine the range of parameters without causing uncomfortable sensations. We chose the method of constant stimuli in a 2AFC paradigm (Kandel et al., 2012) which could reduce the impact of a subject's error of habituation and anticipation compared with the method of minimal change. An important premise was that the subject knew there was definitely a stimulus in one of two intervals within a trial, and he/she was required to choose a preferred one. The detection threshold charge in our finding was about 0.2 μC (1 mA × 0.2 ms = 0.2 μC) lower than 0.6 μC or so for TENS of median or ulnar nerves deep beneath the skin (D'Anna et al., 2017). Under TENS of PFTs, there were no induced strong local sensation of skin or muscle movement happening otherwise for TENS of median or ulnar nerve. By adopting extraneural Cuff or FINE (Flat Interface Nerve Electrode) electrodes, the charge threshold was as small as about 0.1 μC for artificial tactile sensation (Graczyk et al., 2016). Additionally, the maximum charge injected into median and ulnar nerves were 8 and 24 nC using intraneural TIME (Transversal Intrafascicular Multichannel Electrode) electrodes (Raspopovic et al., 2014), and it was also reported that the injected charge threshold ranged from 4.25 nC to 17.5 nC with LIFE (Longitudinal Intrafascicular Electrode) electrodes (Dhillon and Horch, 2005). The detection thresholds in our study were significantly higher than those under invasive circumstances, which indicated that more invasiveness would require less charge to excite the sensory afferents. Since the attention of the subject was engaged in detecting if there existed a stimulus, the detection thresholds in this operational definition might not be detected in other tasks or in daily life, which was possibly due to sensory inputs selection mechanism of attention (Hsiao et al., 1993). Consequently, it was difficult for subjects to describe the perceived intensity near the detection threshold. This was in accordance with the typical response of stimuli near the detection threshold (Flesher et al., 2016). Therefore, the default values of the PA, PW and PF were set a little higher than the corresponding detection thresholds to make sure that the subjects had perceptible and comfortable sensations during experiments of electrical stimulus discrimination and phantom finger recognition.

During the TENS of PFTs, the elicited artificial sensations would convey more information than just magnitude in sensory modalities such as “pressure,” “vibration,” “tingling,” and a variable sensation area. While, for perceived intensity quantification under TENS of PFTs, the subject was instructed to ignore the sensory modality or area changes and only focused on the perceived intensity which was indexed by mechanical indentation depth on the contralateral healthy finger pulp. For participants, the elicited sensations were described as “natural sensation, but they were still different from the sensations under mechanical stimuli.” They described that “the sensation of electrical stimuli is deeper and sharper than feeling under mechanical pressure.” Especially for PF modulation, they felt a little confused to match the intensity of a sharp sting elicited by electrical stimuli with PF above 400 Hz to the mechanical counterparts.

Within the tested stimulus range, the perceived intensities boosted linearly with the increasing PA, and the changing tendencies were similar to the PW modulation in Figure 4. On the other hand, for PF modulation, the intensities were only enhanced linearly with frequencies from 0 to 200 Hz, and remained almost stable for higher frequencies. This was probably due to the reason that the charge per phase was changed under PA and PW stimulation to activate sensory afferents, while the firing rate of fibers changed for the PF modulation (Graczyk et al., 2016). For the PW and lower PF modulations, similar findings existed for peripheral nerve stimulation using FINE or spiral Cuff electrodes. The perceived intensities increased linearly with both PW and PF increasing (Graczyk et al., 2016), where the frequencies were from 25 to 166 Hz. Theoretically, the subjective experience of the perceived intensity was expressed by a power function (Stevens, 1957). For some somatosensory experience, the power function could have a unity exponent which showed a linear relationship (Kandel et al., 2012).

For determination of JNDs, the Weber fraction (Ekman, 1959) was adopted to represent the subjects' abilities to discriminate stimuli. The subjects were required to focus on the difference of perceived intensities between two stimuli in each trial while ignoring other modality or area changes, etc. The smaller the Weber fraction, the better the stimulus discriminability. For the PF modulation, the corresponding Weber fractions were larger than those in PA and PW modulations. Graczyk et al. also denoted that Weber fractions in the PW modulation was much lower than that in PF modulation (Graczyk et al., 2016). The JND for PF was 16.5 ± 1.6 Hz at 50-Hz reference with the Weber fraction of 0.33. The JND for PW was 6.7 ± 1.0 μs, yielding a Weber fraction of 0.05, which was significantly lower than Weber fractions of PF.

The performance in phantom finger recognition without additional training on purpose showed that the main misjudgments were associated with the adjacent PFTs, which could be due to the crosstalk from the electric field spreading during TENS for a specific PFT. Much smaller electrode could be adopted to minimize this kind of misjudgments. There existed incomplete judgment under TENS of more than one PFT. This kind of misjudgment might be due to the masking effect, which meant that the perception of one phantom finger could be also influenced by sensation from other PFTs (Gescheider et al., 1970). Besides, the deteriorated phantom finger recognition could also be resulted from the fact that uniform stimulating current parameters were adopted for tested PFTs among these six subjects with different detection thresholds. The artificial tactile sensation functioned as a process of perception which included “organization, identification and interpretation of sensory information in order to present and to understand the input information, or the environment” (Schacter, 2012). Although there existed some incorrect justice for phantom finger recognition, the discrimination ability of different phantom fingers was empirical, and would be improved through training as a part of learning process (Delhaye et al., 2016; Chai et al., 2017). The recognition of simultaneous stimulation was close to others' work in intracortical sensory feedback, which was 85% for one channel and 53% for two channels. This recognition performance would be advanced by recruiting more and smaller subsets of fibers individually through high electrode density and optimizing stimulating parameters and sites.

In the past several years, the implanted Cuff (Ortiz-Catalan et al., 2014; Tan et al., 2014; Graczyk et al., 2016), USEA (Utah Slanted Electrode Array) (Warwick et al., 2003; Ledbetter et al., 2013), LIFE (Dhillon et al., 2004), and TIME (Boretius et al., 2010; Raspopovic et al., 2014) electrodes were adopted to help produce natural sensation of lost fingers or palms, which made it feasible to accomplish closed-loop motor control of objects in a lab environment (Ortiz-Catalan et al., 2014; Tan et al., 2014; Graczyk et al., 2016). On the other hand, TENS of PFTs by surface electrodes also produced sensation of individual fingers comparable to that for the invasive sensory feedback scheme. However, due to the relatively large surface electrode size and limitation of PFT space, usually one stimulating electrode was located on the MSP within a PFT, and it was hard to stably discriminate different areas within one phantom finger. While, for invasive methods, sensation of some localized areas for a phantom finger could be stably discriminated (Raspopovic et al., 2014; Tan et al., 2014; Graczyk et al., 2016), which would be due to the reason that an implantable microelectrode could supply a more localized stimulation of sensory neurons. In addition, with the number of stimulating electrodes under simultaneous stimulation, recognition of different phantom fingers deteriorated in our study, and the correct ratio decreased from 85.83% (one-channel stimulation) to 67.67% for two channels and 46.44% for four channels. Although the correct ratios were lower for two and four channel stimulation, they were greatly higher than their corresponding chance level as 10 and 6.7%, respectively. In our opinion, the incomplete or partial misjudgment of phantom fingers would partly affect the sensation of object details, but during real-world closed-loop control of prosthetic hands, there existed timing difference of activation among different electrodes (Raspopovic et al., 2014). So more sophisticated encoding approaches introducing this kind of timing difference could be adopted to improve the phantom finger recognition for clinical applications. It was reported that there were roughly 65% of trans-radial amputees with some form of phantom hand sensation (D'Anna et al., 2017). For these amputees, TENS of PFTs would be more appropriate having stable selectivity of individual fingers. For those with high-level amputation and without PFTs, the invasive sensory feedback scheme would be more suitable.

Tactile sensory feedback is undoubtedly essential for the engagement in manipulation and feeling of body ownership of the prosthesis. For now, confusion with the meanings of the resulted artificial sensation and the high cognitive load are still the key issues for the sensory feedback, which requires a more intuitive and high discriminative neural interface (Farina and Amsüss, 2016; Svensson et al., 2017). Others' studies revealed that the phantom finger sensations by mechanical stimulation of the residual stump mapped well to the corresponding normal fingers in the primary somatosensory cortex using fMRI (Björkman et al., 2012). Moreover, our previous work also revealed that the responses related to the phantom finger sensation under TENS were observed in the somatosensory cortex by using MEG neuroimaging technique (Chen et al., 2017). For those reasons, the PFTs under TENS would be intuitive to be recognized and understood by part of the upper-limb prosthetic users. This present work would provide guidelines for strategy selection of artificial tactile feedback in prosthetic hands with less cognitive load for potential clinical applications.

## Conclusion

The discrimination ability of phantom finger sensations elicited by TENS of the PFTs were characterized. We focused on the perceived intensity quantification, electrical stimulus discrimination and phantom finger recognition based on psychophysical experiments. The participants could discern small changes of stimuli in PA, PF, and PW modulations. Although the more number of PFTs under simultaneous stimulation would convey richer tactile information, the recognition performance would deteriorate. Our present studies would shed a light on the optimization of the stimulating strategy to accomplish the clinical application for the intelligent upper-limb prosthetics in the near future. In our future work, we would dig into the objective somatosensory cortical responses objectively by MEG, and further elucidated the neural basis about the discrimination and recognition characteristics.

## Ethics statement

All the psychophysical experiments were carried out in terms of the Declaration of Helsinki, and approved by the Ethics Committee of Human and Animal Experiments at School of Biomedical Engineering, Shanghai Jiao Tong University (No. 2016012). All the subjects or participants were informed of the whole experimental procedure, and signed the informed consent form before experiments.

## Author contributions

XS contributed to the design of the overall psychophysical experiments and data analyses. ML conducted the psychophysical experiments and data analyses. ML wrote the first draft and XS also contributed to the whole manuscript revision. DZ, LH, and YC contributed to subject recruitment and the experiment. XC, YC, and JG were involved in the establishment of the experimental setup. All authors were active in the editing and revising processes of the manuscript. All authors read and approved the final manuscript.

### Conflict of interest statement

LH was employed by company Shanghai Health 51 Net Technology Co., Ltd. The other authors declare that the research was conducted in the absence of any commercial or financial relationships that could be construed as a potential conflict of interest.
